# Genes4Epilepsy: An epilepsy gene resource

**DOI:** 10.1111/epi.17547

**Published:** 2023-03-09

**Authors:** Karen L. Oliver, Ingrid E. Scheffer, Mark F. Bennett, Bronwyn E. Grinton, Melanie Bahlo, Samuel F. Berkovic

**Affiliations:** ^1^ Department of Medicine, Epilepsy Research Centre University of Melbourne, Austin Health Melbourne Victoria Australia; ^2^ Population Health and Immunity Division The Walter and Eliza Hall Institute of Medical Research Parkville Victoria Australia; ^3^ Department of Medical Biology The University of Melbourne Parkville Victoria Australia; ^4^ Florey Institute of Neuroscience and Mental Health Heidelberg Victoria Australia; ^5^ Department of Paediatrics University of Melbourne Melbourne Victoria Australia; ^6^ Murdoch Children's Research Institute Parkville Victoria Australia

**Keywords:** epilepsy panel, genetic architecture, monogenic disease, pathogenic gene resource

## Abstract

**Objective:**

“How many epilepsy genes are there?” is a frequently asked question. We sought to (1) provide a curated list of genes that cause monogenic epilepsies, and (2) compare and contrast epilepsy gene panels from multiple sources.

**Methods:**

We compared genes included on the epilepsy panels (as of July 29, 2022) of four clinical diagnostic providers: Invitae, GeneDx, Fulgent Genetics, and Blueprint Genetics; and two research resources: PanelApp Australia and ClinGen. A master list of all unique genes was supplemented by additional genes identified via PubMed searches up until August 15, 2022, using the search terms “genetics” AND/OR “epilepsy” AND/OR “seizures”. Evidence supporting a monogenic role for all genes was manually reviewed; those with limited or disputed evidence were excluded. All genes were annotated according to inheritance pattern and broad epilepsy phenotype.

**Results:**

The comparison of genes included on epilepsy clinical panels revealed high heterogeneity in both number of genes (range: 144–511) and content. Just 111 genes (15.5%) were included on all four clinical panels. Subsequent manual curation of all “epilepsy genes” identified >900 monogenic etiologies. Almost 90% of genes were associated with developmental and epileptic encephalopathies. By comparison only 5% of genes were associated with monogenic causes of “common epilepsies” (i.e., generalized and focal epilepsy syndromes). Autosomal recessive genes were most frequent (56% of genes); however, this varied according to the associated epilepsy phenotype(s). Genes associated with common epilepsy syndromes were more likely to be dominantly inherited and associated with multiple epilepsy types.

**Significance:**

Our curated list of monogenic epilepsy genes is publicly available: github.com/bahlolab/genes4epilepsy and will be regularly updated. This gene resource can be utilized to target genes beyond those included on clinical gene panels, for gene enrichment methods and candidate gene prioritization. We invite ongoing feedback and contributions from the scientific community via genes4-epilepsy@unimelb.edu.au.

**Key points**

- The number of monogenic genes associated with epilepsy has risen exponentially in the last decade;
- There remains great disparity in genes included on different clinical and/or research gene panels;
- We identify >900 monogenic “epilepsy genes,” with ~90% associated with developmental and epileptic encephalopathies;
- Inheritance patterns vary for different epilepsy phenotypes;
- Our curated list of monogenic epilepsy genes is publicly available from: github.com/bahlolab/genes4epilepsy and will be updated half yearly.

## 1 INTRODUCTION

Prior to the advent of high‐throughput sequencing technologies, it was possible to memorize the names of every well‐established monogenic epilepsy gene; now there is an “alphabet soup” of hundreds of genes. This explosion in knowledge has been driven by major technological advances that have revolutionized our understanding of the genetics of human disease more broadly.

**FIGURE 1:**
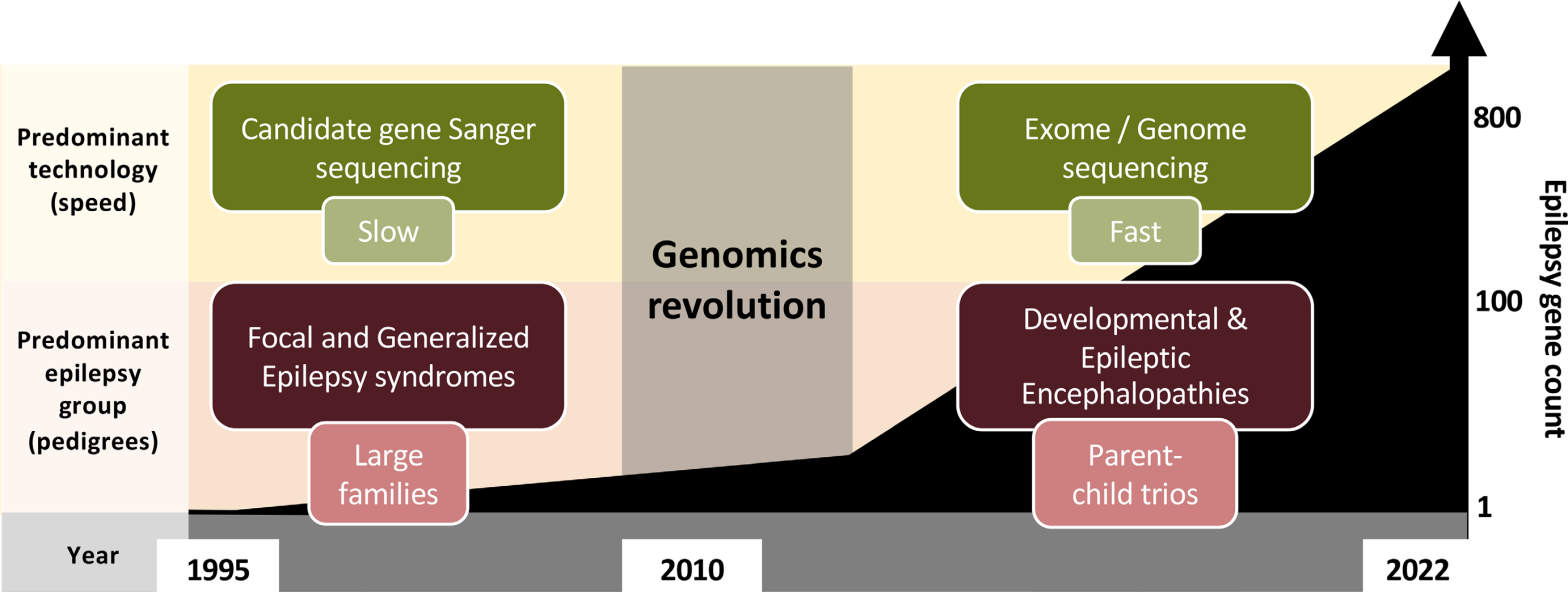
Approximate timeline and impact of monogenic epilepsy gene discoveries over the last 27 years.

With the discovery of *CHRNA4* in 1995,^1^ the era of candidate gene Sanger sequencing in the epilepsies began, and the road to each gene breakthrough took several years (Figure 1). Success relied upon the meticulous clinical workup of rare large multiplex families, with focal or generalized epilepsy syndromes segregating in a highly penetrant manner through multiple generations. Such families were amenable to linkage analysis and were critical for targeting expensive, laborious candidate gene sequencing efforts. With one or two new discoveries per year, curating the list of “epilepsy genes” was a manageable task for the first 15 or so years. The release of the human genome reference sequence in the early 2000s^2^ was revolutionary in providing an important base for future high‐throughput genomic sequencing platform development and subsequent gene discovery.

This genomics revolution saw a transition away from candidate gene Sanger sequencing as the predominant gene discovery methodology to whole exome (and eventually genome) sequencing technology. As a result, we have witnessed an exponential growth in the discovery of monogenic epilepsy genes or “genes of major effect.” The impact of this has been greatest in patients with the most severe group of epilepsies, the developmental and epileptic encephalopathies (DEEs) (Figure 1). Here, the application of high‐throughput sequencing technology to parent–child trios proved particularly powerful for detecting de novo dominant variants and, more recently, biallelic recessive variants. In fact, there is a growing list of genes for both neurodevelopmental disorders (NDDs) and DEEs, with disease‐causing variants under both autosomal dominant and recessive inheritance models.

With multiple new genes being published *per week*, compared to *per year* previously, keeping up to date with the number of “epilepsy genes” has become an almost insurmountable task. It is, however, critical to ensure that all known genes are scrutinized for pathogenic variants to determine the etiology in individuals with epilepsy. Epilepsy gene panel analyses have become routine diagnostic tests in clinical practice in many regions, particularly for patients with severe childhood‐onset epilepsies. Many clinical diagnostic providers maintain their own version of an “epilepsy gene panel” that vary significantly in the genes that are interrogated.^3^, ^4^ Important efforts to provide clinical‐grade gene lists are being made by both ClinGen^5^ and PanelApp,^6^ where experts review and grade each putative epilepsy gene according to strict criteria. Unsurprisingly the review process can be slow and lag the fast‐emerging literature, which could lead to important missed epilepsy genetic diagnoses.

In the research setting, gene lists are regularly used for nonclinical gene panel applications, genetic enrichment analyses, and candidate gene prioritization efforts. For example, understanding the collective characteristics of epilepsy genes can help inform future gene discoveries and has the potential to reveal important shared biological features. Already, just appreciating the large number of monogenic genes associated with epilepsy sets it apart from other complex diseases where the number of Mendelian causes is far fewer.^7^

Here, we collate the growing list of monogenic genes (as of August 15, 2022) that, if mutated, have been reported to cause epilepsy. We stratify genes by inheritance model and broad epilepsy phenotype(s). We aim to include all monogenic genes associated with phenotypes where epilepsy is the primary clinical presentation, but also include NDDs, malformation, and metabolic genes that may cause seizures in only a subset of patients. We provide our epilepsy gene list at github.com/bahlolab/genes4epilepsy; it is version controlled and will be updated biannually. Maintaining such a list as a research group with an established track record is critical for our ongoing epilepsy genetic research. Furthermore, we invite and welcome feedback and contributions from the epilepsy genetics community via genes4-epilepsy@unimelb.edu.au.

## 2 MATERIALS AND METHODS

### 2.1 Comparison of epilepsy gene panels

To appreciate the current variability in clinical testing panels provided by different genetic services we downloaded and cross‐referenced the epilepsy lists from four commonly used diagnostic laboratories: Invitae, GeneDx, Fulgent Genetics, and Blueprint Genetics. We further compared these lists with the epilepsy genes that have met the respective inclusion criteria for PanelApp Australia and ClinGen research panels (Table 1).

**TABLE 1:** Diagnostic and research epilepsy gene panels.

Provider	Service	Panel name	Website	Date accessed
Invitae	Clinical	Epilepsy panel (primary)	https://www.invitae.com/en/providers/test‐catalog/test‐03401	29‐Jul‐2022
GeneDx	Clinical	Comprehensive epilepsy panel	https://www.genedx.com/tests/detail/comprehensive‐epilepsy‐panel‐317	29‐Jul‐2022
Fulgent Genetics	Clinical	Epilepsy comprehensive NGS panel	https://www.fulgentgenetics.com/epilepsy‐comp	29‐Jul‐2022
Blueprint Genetics	Clinical	Comprehensive epilepsy panel	https://blueprintgenetics.com/tests/panels/neurology/comprehensive‐epilepsy‐panel/	29‐Jul‐2022
PanelApp Australia	Research	Genetic epilepsy (included “green” genes)^a^	https://panelapp.agha.umccr.org/panels/202/	29‐Jul‐2022
ClinGen	Research	Epilepsy Expert Panel (excluded “disputed” or “refuted” genes)^b^	https://search.clinicalgenome.org/kb/affiliate/10005?page=1&size=25&search=	29‐Jul‐2022

Abbreviation: NGS, next generation sequencing.

^a^ PanelApp “green genes” should be a conservative (diagnostic‐grade) set of genes.^25^

^b^ ClinGen reviewers classify each gene as “definitive,” “strong,” “moderate,” “limited,” “disputed,” or “refuted.”

### 2.2 Curation of monogenic epilepsy genes

To create a comprehensive list of monogenic epilepsy genes we combined all clinical and research lists (Table 1). The list was supplemented by the addition of genes identified via weekly automated PubMed searches from March 30, 2013 to August 15, 2022, using PubCrawler (https://pubcrawler.gen.tcd.ie/) with search terms “genetics” AND/OR “epilepsy” AND/OR “seizures”. Furthermore, we added individual genes referred to us by research scientists via direct communication.

All genes were assessed manually for a clear association with an epilepsy phenotype in at least two families where reported variants met American College of Medical Genetics guidelines for “likely pathogenic” / “pathogenic”^8^; rare exceptions were made for single families if published with strong functional support.

Genes were then coded into *at least one* of the following genetic inheritance and broad clinical groups:

1. Inheritance pattern: autosomal dominant (AD), autosomal recessive (AR), X‐linked (XL), mitochondrial (MT);
2. Broad phenotypic group: genetic generalized epilepsy (GGE), focal epilepsy, DEE, progressive myoclonus epilepsy (PME), malformations of cortical development (MCDs).

Of note, limited clinical data often prevent a clear distinction between NDD and DEE being made, thus metabolic syndrome and NDD genes were classified as “DEE” due to their overlapping phenotypes. Genes associated with familial adult myoclonic epilepsy (FAME) were classified as “PME.” The list of curated “epilepsy genes” does not include:

- Epilepsy‐associated copy number variants (CNVs) or the candidate genes they encompass.
- Candidate genes reported by genome‐wide association studies (GWASs).
- Genes associated with non‐epileptic paroxysmal events (e.g., hyperekplexia).
- Brain somatic pathogenic variant genes unless germline pathogenic variants of the same gene have also been reported.

## 3 RESULTS

### 3.1 Comparison of epilepsy gene panels

**FIGURE 2:**
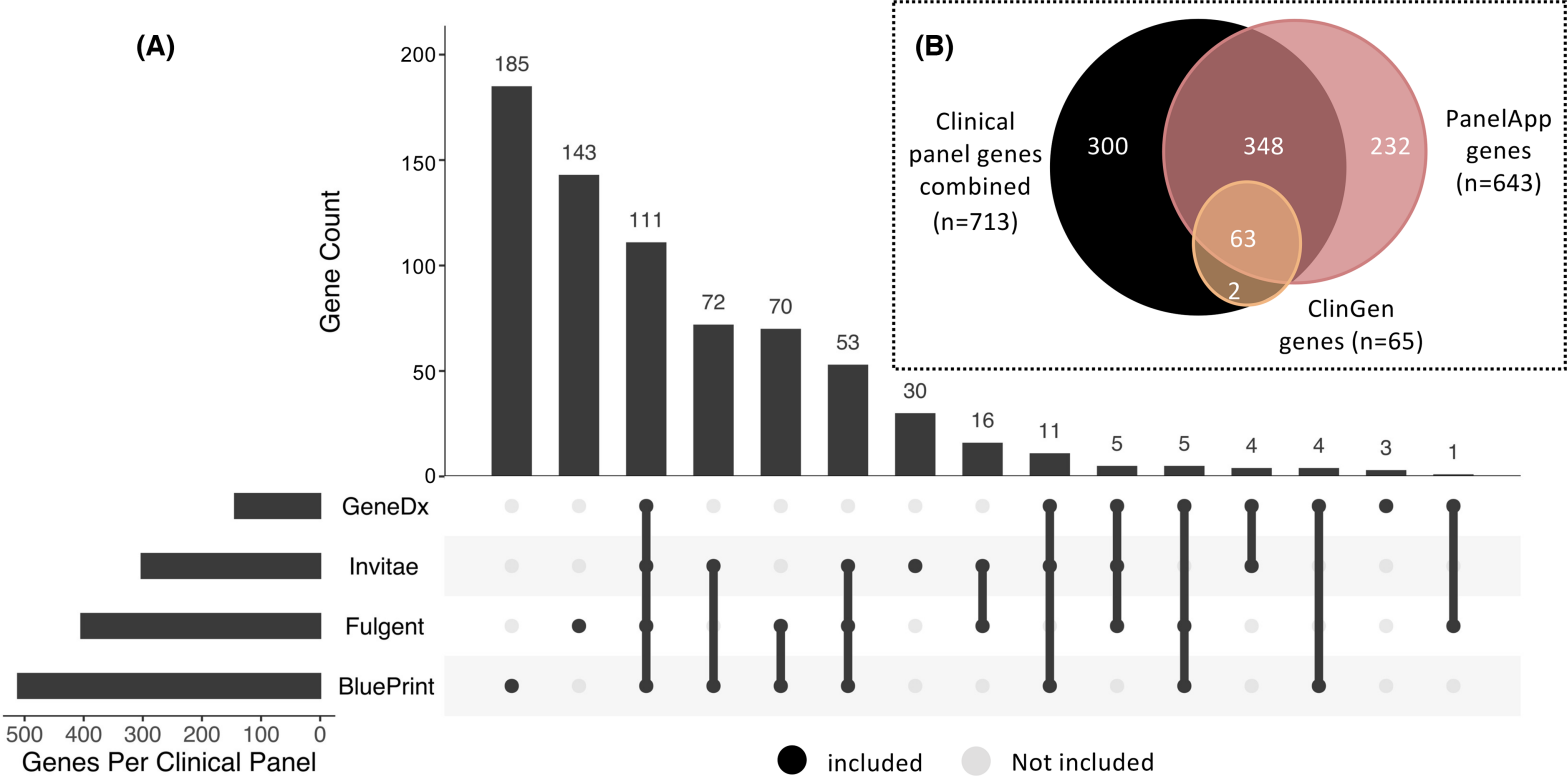
(A) UpSet plot^26^, ^27^ summarizing the intersection of the 713 epilepsy genes across all four clinical panels. Black dots indicate that a panel is part of an intersection with connecting lines indicating the direction the plot should be read. The numbers of genes captured by each intersection are plotted above as a bar chart (e.g., 185/713 genes are unique to the BluePrint panel and 70/713 genes are included on the Fulgent and BluePrint panels, but not on GeneDx or Invitae). (B) Venn diagram summarizing the overlap for the 945 unique epilepsy genes included on either at least one of four clinical panels and/or the two research panels (PanelApp or ClinGen).

Diagnostic panel sizes ranged from 144 (GeneDx) to 511 (BluePrint Genetics) epilepsy genes (mean = 340). Of the total 713 unique genes included on at least one clinical diagnostic panel, only 111 (15.5%) were present on all four panel lists (Figure 2A), highlighting the high level of discordance between lists. The two research panels captured only a small portion of the clinical panel genes (ClinGen, *n* = 65) or, as the largest list, PanelApp (*n* = 643), contributed another 232 unique genes (Figure 2B).

### 3.2 Monogenic epilepsy genes and insights

**FIGURE 3:**
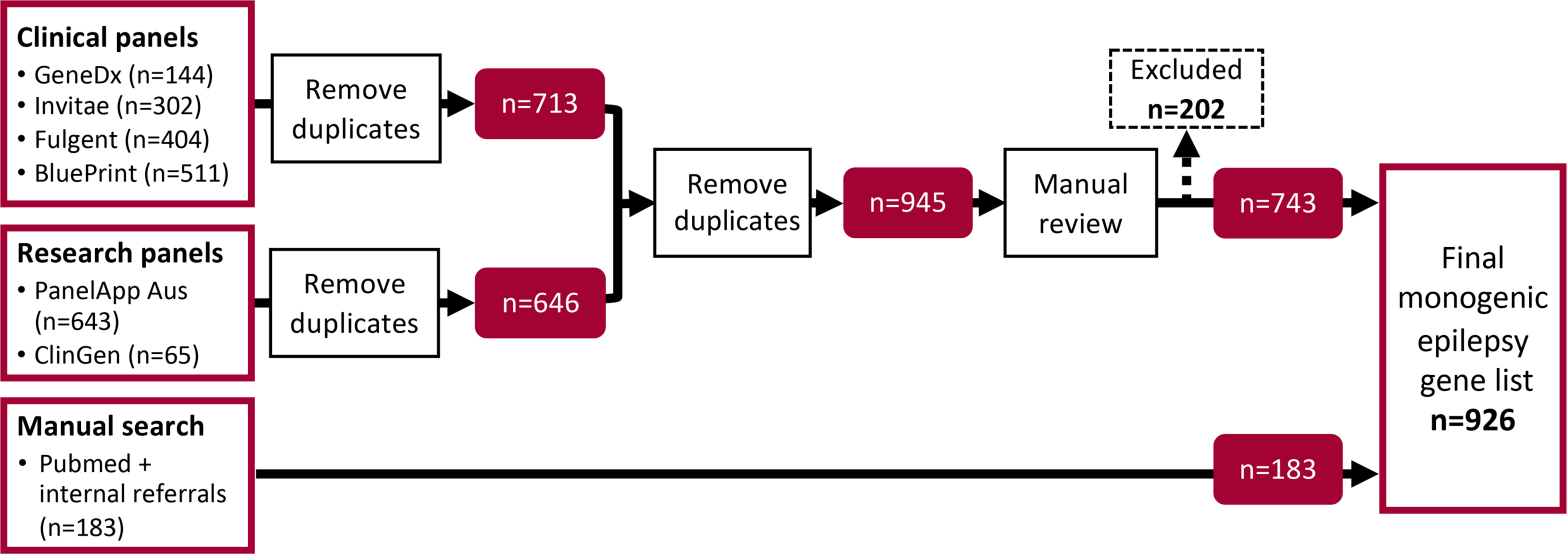
Epilepsy gene list curation workflow.

We curated a list of 926 monogenic epilepsy genes from the 1128 genes reviewed (Figure 3; Table S1). The remaining 202 genes were excluded due to insufficient or disputed evidence (Figure 3; Table S2).

**FIGURE 4:**
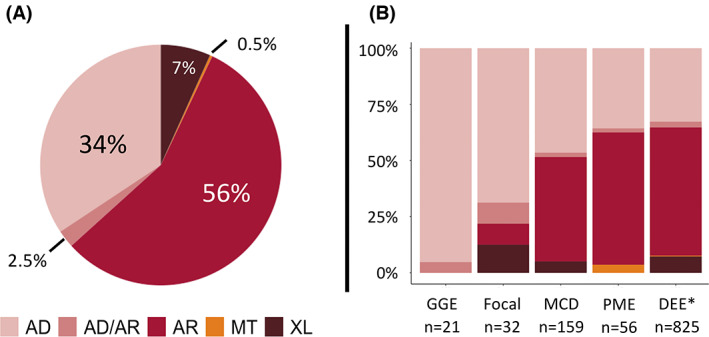
(A) Overall percentage breakdown of gene inheritance patterns. (B) Inheritance pattern breakdown by phenotypic group. AD, autosomal dominant; AD/AR, dominant/recessive; AR, autosomal recessive; DEE, developmental and epileptic encephalopathy; GGE, genetic generalized epilepsy; MCD, malformation of cortical development; MT, mitochondrial; NDD, neurodevelopmental disorder; PME, progressive myoclonus epilepsy; XL, X‐linked. *DEE includes NDD and metabolic genes that may only cause seizures in a subset of patients.

More than half (56%) of the curated monogenic epilepsy genes follow autosomal recessive inheritance (Figure 4A). Many of these genes are very rare causes of epilepsy; therefore, this does not necessarily translate to more than half the patients with molecular diagnoses having recessive inheritance. Notably, the inheritance patterns differ according to the broad clinical groups with which the genes are associated (Figure 4B). For example, the “common” GGE and focal epilepsies are more likely to be associated with autosomal dominant genes, whereas DEE and PME genes are predominantly autosomal recessive. Genes associated with MCDs are equally likely to be autosomal dominant or recessive.

Although the PMEs have long been conceptualized as autosomal recessive diseases, the increasing genetic overlap with DEE phenotypes has seen an increase in the number of associated dominant genes (e.g., *KCNC1, DHDDS, NUS1*).^9^ De novo dominant DEE genes, on the other hand, led the initial explosion in monogenic epilepsy gene discoveries. However, metabolic DEE diseases are frequently due to autosomal recessive inheritance^10^ and the number of newly reported recessive genes with overlapping NDD and DEE phenotypes are rising. This is due to the intensive study of patients from inbred populations and more efficient methods to identify biallelic compound heterozygous variants in outbred cases.

X‐linked genes, mitochondrial genes, and genes that cause disease under both autosomal recessive and dominant inheritance models make up a small portion of genes overall (10%). In the case of the latter group, this may be the result of different variant effects. For example, recessive disease might result from two loss of function variants, whereas dominant disease may result from a single gain of function variant in the same gene.^11^, ^12^, ^13^

Almost 90% of the curated monogenic genes have been associated with a DEE phenotype (*n* = 825/926 total). By comparison, just 5% of all epilepsy genes were associated with a GGE and/or focal epilepsy phenotype (*n* = 45/926). The majority of these 45 “common epilepsies” genes are pleiotropic (38/45; 84%). Such pleiotropic epilepsy genes have been associated with multiple epilepsy types, including, but not limited to, DEEs (Table 2).

**TABLE 2:** “Common epilepsy” monogenic genes.

“Common epilepsy” group	Other clinical group(s)	N genes	“Common epilepsy” monogenic genes (i.e., GGE and focal epilepsy)^a^
GGE	GGE only	1	*HCN2*
	GGE, DEE	10	*HCN1, GABRD, CHD4, RORB, GABRG2, GABRB2, GABRA5, KCNC2, CUX2, GABRA1*
	GGE, DEE, PME	2	*CHD2, CACNA1A*
GGE, Focal	GGE, Focal, DEE	7	*SLC6A1, SCN2A, SCN1B, SLC32A1, SCN1A, STX1B, SLC2A1*
	GGE, Focal, DEE, PME	1	*KCNA2*
Focal	Focal only	6	*LGI1, CHRNA4, CHRNB2, CHRNA2, VPS13A, PRIMA1*
	Focal, MCD	6	*TSC1, TSC2, NPRL3, NPRL2, RELN, PIK3C2B*
	Focal, DEE	8	*KCNT1, ARHGEF9, PCDH19, KCNQ2, KCNQ3, SYN1, PRRT2, CNKSR2*
	Focal, DEE, MCD	2	*DEPDC5, GRIN2A*
	Focal, DEE, PME	2	*TBC1D24, SCN8A*

^a^ See Table S3 for relevant references.

## 4 DISCUSSION

We have curated a list of >900 genes causing monogenic disorders associated with epilepsy. In doing so, we determined the current degree of variability in gene panel lists across clinical diagnostic providers. Less than 16% of genes were concordant across all diagnostic clinical panels, which is consistent with previous, similar observations^3^, ^4^ and highlights the challenges in keeping pace with the fast‐evolving field of epilepsy genetics. The issue is not simply new genes being published on a weekly basis, but also newly associated phenotypes and, sometimes, novel patterns of inheritance. Capturing these new findings is important for variant interpretation and diagnosis, but clearly an enormous task, a task we address with this new monogenic epilepsy gene resource. This study will serve as a benchmark now regarding substantiated epilepsy genes in 2022, and our resource will provide a means for clinicians and researchers to identify recently discovered genes in the future in a timely manner.

Periodic reanalysis of molecular data from unsolved patients is essential. It is a highly effective way of improving diagnostic yield due to the inclusion of new disease genes that were not known previously or were not included in the gene panel analyzed previously.^14^, ^15^, ^16^ A curated, up‐to‐date resource with changes tracked over time is ideal for this purpose, enabling clear documentation of the genes analyzed and facilitating easy comparisons between studies. For genome‐wide analyses, this gene list will facilitate prioritization of novel variants by also providing the clinical associations and inheritance models for each gene.

Most epilepsy genes discovered to date are associated with a DEE phenotype. The success in uncovering the hundreds of monogenic genes associated with this rarer group of epilepsies, in addition to the PMEs and MCDs, stands in stark contrast to that seen for common focal and generalized epilepsies. Although more than 50% of patients with a DEE^17^ and up to 80% of patients with a PME^18^ are currently genetically “solved” by finding a pathogenic variant in an established monogenic epilepsy gene, the same currently holds true for only a small minority of patients with a common form of epilepsy. Of interest, large case–control burden studies show molecular overlap between the DEEs and GGE, as they find that rare variants in DEE genes are enriched in patients with GGE.^19^, ^20^ Furthermore, candidate GGE genes implicated in the most recent International League Against Epilepsy (ILAE) GWAS are enriched for established monogenic (mostly DEE) epilepsy genes.^21^

Not surprisingly then, of the small number of monogenic GGE and focal epilepsy genes (5% of the total resource), most are pleiotropic—a term indicating that the gene may influence more than one trait. All except one gene associated with GGE has been associated with at least a DEE phenotype as well (Table 2). Focal epilepsy genes also demonstrate pleiotropy; however, a larger portion are phenotype‐specific compared to GGE. This is consistent with the two largest epilepsy clinical groups having different underlying genetic architectures, as has been noted previously.^20^, ^22^, ^23^

A gene list resource such as this will never be complete or 100% accurate, as the landscape is constantly evolving. Interpreting the literature and curating genes is fraught with human bias and it is unlikely that a true consensus would ever be reached. Furthermore, there is often limited clinical (epileptology) data included in initial gene discovery papers. This meant that we were unable to determine whether all genes met the ILAE syndrome classifications^24^ for each of the epilepsy phenotypes they were grouped under. As a result, we used a particularly broad definition for our DEE clinical group.

In contrast, efforts made by ClinGen, for example, to carefully weight and grade clinical and experimental evidence are highly commended. However, as evidenced by that resource contributing the smallest external epilepsy panel list, the number of genes requiring assessment only continues to grow and outpace the review process. Although the genes listed by our resource have clearly not been reviewed as comprehensively, we have taken a pragmatic view and tractable approach to the ever‐increasing pace of gene discovery. We feel this is a valuable adjunctive strategy, as our intention is not to provide a list of clinical grade genes, but rather a resource for clinicians and researchers working in epilepsy, where the power is in both the number of genes captured and the quality of evidence for those genes, which is still high.

The rate of monogenic discovery is not yet leveling off but must do so eventually as there is a finite number of genes. Then our task will turn to unraveling oligogenic contributions to epilepsy risk for which this resource will also prove useful.

## AUTHOR CONTRIBUTIONS

Resource data curation and maintenance: KLO, MFB, BEG; resource design and conceptualization: all authors; wrote manuscript: KLO; reviewed manuscript: all authors.

## FUNDING INFORMATION

The authors gratefully acknowledge support provided by the Australian National Health and Medical Research Council (NHMRC) Centre for Research Excellence Grant [GNT2006841] and NHMRC Synergy Grant [GNT2010562]. IES is also supported by an NHMRC Senior Investigator Grant [GNT1172897], MB by an NHMRC Senior Investigator Grant [APP1195236], and SFB by an NHMRC Senior Investigator Grant [APP196637]. KLO is supported by the Australian Commonwealth Government and the University of Melbourne Australian Government Research Training Program Scholarship [APP533086]. This work was also supported by the Victorian Government's Operational Infrastructure Support Program, the NHMRC Independent Research Institute Infrastructure Support Scheme (IRIISS).

## CONFLICT OF INTEREST STATEMENT

SFB has received unrestricted educational grants from UCB Pharma, Chiesi, Liva Nova, and Seer Medical; and personal fees from Sequirus and Praxis during the conduct of this study. Ingrid Scheffer has served on scientific advisory boards for BioMarin, Chiesi, Eisai, Encoded Therapeutics, GlaxoSmithKline, Knopp Biosciences, Nutricia, Rogcon, Takeda Pharmaceuticals, UCB, Xenon and Pharmaceuticals; has received speaker honoraria from GlaxoSmithKline, UCB, BioMarin, Biocodex, Chiesi, Liva Nova, Nutricia, Zuellig Pharma, and Eisai; has received funding for travel from UCB, Biocodex, GlaxoSmithKline, Biomarin, and Eisai; has served as an investigator for Anavex Life Sciences, Cerecin Inc, Cerevel Therapeutics, Eisai, Encoded Therapeutics, EpiMinder Inc, Epygenyx, ES‐Therapeutics, GW Pharma, Marinus, Neurocrine BioSciences, Ovid Therapeutics, Takeda Pharmaceuticals, UCB, Ultragenyx, Xenon Pharmaceuticals, Zogenix, and Zynerba; has consulted for Care Beyond Diagnosis, Epilepsy Consortium, Atheneum Partners, Ovid Therapeutics, UCB, Zynerba Pharmaceuticals, BioMarin, Encoded Therapeutics, and Biohaven Pharmaceuticals; and is a Non‐Executive Director of Bellberry Ltd and a Director of the Australian Academy of Health and Medical Sciences and the Australian Council of Learned Academies Limited. None of the remaining authors has any conflict of interest to disclose.

## IRB APPROVAL

Not applicable.

## Supporting information


Table S1–S3

Data S1
