# Supplementary material for: Genes4Epilepsy: An epilepsy gene resource
Source: Epilepsia. 2023 Mar 9;64(5):1368–75. doi: 10.1111/epi.17547 (PMC10952165; doi:10.1111/epi.17547)
Supplement: Supplementary file 2 — Data S1 [file EPI-64-1368-s001.docx]

**Supplementary Table 1. List of 926 monogenic epilepsy genes. Please visit github.com/bahlolab/genes4epilepsy for current list.**

**Supplementary Table 2. List of 202 genes excluded from monogenic epilepsy gene list due to insufficient or disputed evidence at the time of this publication.**

**Supplementary Table 3. References for "common epilepsy" monogenic genes listed in Table 2.**
